# A Satellite View of Pollution on the Ground: Long-Term Changes in Global Nitrogen Dioxide

**DOI:** 10.1289/ehp.124-A56

**Published:** 2016-03-01

**Authors:** Nate Seltenrich

**Affiliations:** Nate Seltenrich covers science and the environment from Petaluma, CA. His work has appeared in *High Country News*, *Sierra*, *Yale Environment 360*, *Earth Island Journal*, and other regional and national publications.

From monitoring harmful algal blooms to predicting the spread of infectious diseases, recent advances in satellite-based remote sensing have been a boon to environmental health science.^1^ In particular, the capacity to assess air quality from space is built on decades of work now beginning to pay dividends. This issue of *EHP* includes the first global estimate of long-term changes in ground-level nitrogen dioxide (NO_2_), offering a new tool for evaluating how land-use and policy decisions influence pollution levels on a large scale.^2^

NO_2_ is one of six criteria pollutants regulated by the U.S. Environmental Protection Agency.^3^ Generated primarily from vehicle exhaust, but also by power plants and heavy equipment, the compound causes a variety of adverse respiratory effects and contributes to the formation of ozone, another harmful criteria pollutant.^4^

**Figure:**
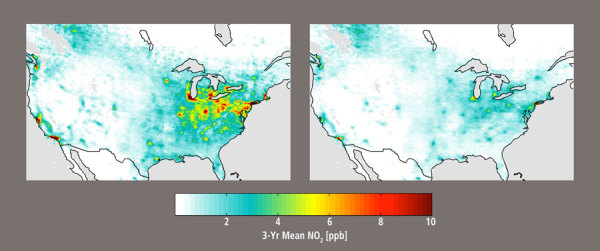
Remotely sensed data indicate that levels of NO_2_ decreased in North America and other high-income regions between 1996 (left) and 2012 (right), likely as a result of assorted policy and economic factors. Other areas fared worse, with levels in industrialized China more than doubling. Source: Geddes (2016)^2^

Ground-level monitors keep close tabs on NO_2_, but they are located primarily in urban and industrial areas—in those regions where they exist at all. Remote sensing promises to fill the gaps in monitoring data because satellites can “see” areas outside the reach of ground-level monitors.

“We’re trying to account for the fact that there are lots of places around the world where there hasn’t historically been a lot of air quality data,” says lead author Jeff Geddes, a postdoctoral fellow at Dalhousie University in Halifax. Coauthor and lab leader Randall Martin notes that while satellite readings can augment ground-based air quality monitoring, they’re not designed as a replacement; ground-based observations provide critical reference points, he says, while remote sensing measurements enable extrapolation to areas without ground data.

The first demonstration of inferring ground-level NO_2_ levels from a satellite—in this case using a single instrument over North America for a single year—came in 2008.^5^ Eight years later another team that includes two of the same researchers has merged data from three satellites covering the entire planet for 17 years. The new analysis suggests that population-weighted mean concentrations of NO_2_ in high-income regions of North America, Western Europe, and Asia Pacific decreased by 50%, 30%, and 20%, respectively, between 1996 and 2012.^2^ This is likely attributable to a mix of emissions controls, changes in transportation infrastructure, and broader economic trends such as the 2008 recession.^6^^,^^7^^,^^8^^,^^9^^,^^10^^,^^11^ East Asia, predominately represented by an industrialized and rapidly developing China, saw levels increase 2.7 times over the same time span.

By overlaying population data on the pollution map, the authors found that during the study period approximately 1.6 billion people lived in regions where average NO_2_ significantly decreased, versus 3.2 billion who lived where levels significantly increased.^2^ “You can see on a broad scale how we’ve sort of just shifted this problem around,” says Jeffrey Brook, an assistant professor of environmental health at the University of Toronto and senior research scientist for the Canadian government. Brook was not involved with the study but has collaborated with members of the Dalhousie team in the past.

Aaron Cohen, a principal scientist with the Health Effects Institute in Boston, says the study results largely agree with existing data on long-term trends for fine particulate matter (PM_2.5_), which indicate increases and decreases over time in some of the same parts of the world.^12^ “The estimates that [the authors] present, which I find very interesting, are corroborating global trends in combustion-related air pollution,” he says. Although Cohen considers PM_2.5_ a better overall indicator for estimating the human health burden of ambient air pollution, he says NO_2_ estimates can provide important information about specific sources of pollution, such as vehicle and diesel emissions in urban areas.

Because of current inherent limitations to remote sensing, the new paper does not provide the sort of reliable absolute values needed for some epidemiological research. In the coming years, the European Space Agency’s Tropospheric Monitoring Instrument (TROPOMI)^13^ and NASA’s TEMPO mission^14^ will further improve researchers’ ability to assess air quality from space. “A lot of great progress has been made with remote sensing, but it certainly hasn’t finished,” Martin says. “There’s a lot more work that needs to be done.”
